# Using Signal Detection Theory to Better Understand Cognitive Fatigue

**DOI:** 10.3389/fpsyg.2020.579188

**Published:** 2021-01-15

**Authors:** Glenn R. Wylie, Bing Yao, Joshua Sandry, John DeLuca

**Affiliations:** ^1^Kessler Foundation, Rocco Ortenzio Neuroimaging Center, Kessler Foundation, West Orange, NJ, United States; ^2^Department of Physical Medicine and Rehabilitation, New Jersey Medical School, Newark, NJ, United States; ^3^The Department of Veterans’ Affairs, The War Related Illness and Injury Study Center, New Jersey Healthcare System, East Orange Campus, East Orange, NJ, United States; ^4^Psychology Department, Montclair State University, Montclair, NJ, United States; ^5^Department of Neurology, New Jersey Medical School, Newark, NJ, United States

**Keywords:** signal detection theory, fMRI, cognitive fatigue, working memory, striatum

## Abstract

When we are fatigued, we feel that our performance is worse than when we are fresh. Yet, for over 100 years, researchers have been unable to identify an objective, behavioral measure that covaries with the subjective experience of fatigue. Previous work suggests that the metrics of signal detection theory (SDT)—response bias (criterion) and perceptual certainty (*d*’)—may change as a function of fatigue, but no work has yet been done to examine whether these metrics covary with fatigue. Here, we investigated cognitive fatigue using SDT. We induced fatigue through repetitive performance of the *n*-back working memory task, while functional magnetic resonance imaging (fMRI) data was acquired. We also assessed cognitive fatigue at intervals throughout. This enabled us to assess not only whether criterion and *d*’ covary with cognitive fatigue but also whether similar patterns of brain activation underlie cognitive fatigue and SDT measures. Our results show that both criterion and *d*’ were correlated with changes in cognitive fatigue: as fatigue increased, subjects became more conservative in their response bias and their perceptual certainty declined. Furthermore, activation in the striatum of the basal ganglia was also related to cognitive fatigue, criterion, and *d*’. These results suggest that SDT measures represent an objective measure of cognitive fatigue. Additionally, the overlap and difference in the fMRI results between cognitive fatigue and SDT measures indicate that these measures are related while also separate. In sum, we show the relevance of SDT measures in the understanding of fatigue, thus providing researchers with a new set of tools with which to better understand the nature and consequences of cognitive fatigue.

## Introduction

Fatigue resulting from cognitive work (cognitive fatigue) is a common experience, caused by tasks that require care and skill such as air traffic control (Orasanu et al., 2012; Kuo et al., 2017) or driving (Matthews and Desmond, 2002). Furthermore, cognitive fatigue is a common sequela following brain injury [e.g., traumatic brain injury (TBI) or stroke] or disease [e.g., multiple sclerosis (MS) or Parkinson’s disease]. Intuitively, we feel that performance should decline as cognitive fatigue increases, yet a large body of research shows that this is not the case (Craig and Cooper, 1992; Stoner, 1996; Torres-Harding and Leonard, 2005). The disappointing lack of correlation between the subjective feelings of cognitive fatigue and objective measures of performance such as response time (RT) and accuracy has hampered research in this area. However, fatigue has been linked to decrements in perceptual sensitivity [i.e., a reduced ability to distinguish stimuli requiring a response (targets) from stimuli that do not require a response (non-targets)]—or *d*’, a measure derived from signal detection theory (SDT) (Green and Swets, 1966; Lynn and Barrett, 2014)—in the human factors literature (Matthews and Desmond, 2002), which may be linked to well-documented decrements in *d*’ associated with vigilance tasks (See et al., 1995). For example, Matthews and Desmond (2002) found that perceptual sensitivity was reduced and fatigue was increased following a difficult “drive” in a driving simulator (relative to an easier drive). Thus, while simple RT and accuracy correlate poorly with fatigue, the tools of SDT (and perceptual sensitivity in particular) may provide better objective indices of fatigue. However, while decrements in *d*’ have been demonstrated after fatigue has been induced (i.e., before vs. after fatigue induction), it has not been shown that progressive increases in fatigue are associated with progressive decreases in perceptual sensitivity. Showing a correlation of this sort between *d*’ and fatigue would provide researchers with a powerful tool to better understand fatigue.

While perceptual sensitivity (*d*’) has been shown to be worse after fatigue induction (Matthews and Desmond, 2002), the effect of fatigue on bias (β), or criterion, which is the other main SDT measure, has not been investigated. In the context of SDT, criterion refers to the amount of evidence one requires before releasing a response: a liberal criterion means that one requires relatively little evidence that a stimulus is a target before releasing a response; a conservative criterion means that one requires relatively more evidence before releasing a response. It is somewhat surprising that changes in criterion have not been investigated in the fatigue literature since recent investigations into fatigue have suggested that fatigue reflects, at least in part, a change in the balance between effort and reward (Dobryakova et al., 2015; Wylie et al., 2017b; Massar et al., 2018; Müller and Apps, 2018). Signal detection theory predicts that changes in the payoff matrix—that is, the balance between effort and reward—will be reflected in changes in criterion. It has been repeatedly shown that changing the payoff matrix by increasing the reward subjects receive reduces fatigue (Matthews and Desmond, 2002; Boksem et al., 2006; Lorist et al., 2009), but hitherto, there have been no investigations into whether changes in fatigue are correlated with changes in criterion.

In the work described here, we induced fatigue by asking subjects to repeatedly perform two conditions of the *n*-back working memory task: the 0-back condition and the 2-back condition (Wylie et al., 2017a, b, 2018). By using the accuracy on different types of trials (correct rejections and false alarms), we calculated subjects’ sensitivity and their response bias, using SDT (Green and Swets, 1966; Lynn and Barrett, 2014). Furthermore, at baseline, and after each of the eight runs of the tasks, we assessed subjects’ cognitive fatigue using the visual analog scale of fatigue (VAS-F) (Shahid et al., 2011). This design allowed us to assess whether changes in perceptual sensitivity and criterion were correlated with subjective reports of cognitive fatigue. Finally, both structural and functional magnetic resonance imaging (fMRI) data were acquired while subjects performed the tasks. This allowed us to assess whether brain areas that were sensitive to changes in cognitive fatigue were also sensitive to changes in perceptual certainty and/or criterion. Based on the literature (Chaudhuri and Behan, 2004), and on our previous work (Dobryakova et al., 2017), we hypothesized that the striatum of the basal ganglia would play a central role. Several studies, both from our lab (e.g., Dobryakova et al., 2013, 2017; Wylie et al., 2017a) and from others (e.g., Chaudhuri and Behan, 2004; Tang et al., 2013; Nakagawa et al., 2016), have indicated that the striatum in general and the caudate in particular are implicated in fatigue. The role of the striatum was assessed both from a structural standpoint—investigating whether the volume of the striatum covaried with cognitive fatigue and SDT measures—and from a functional standpoint—investigating whether activation in the striatum covaried with cognitive fatigue and SDT measures.

## Materials and Methods

### Subjects

Forty-eight healthy volunteers participated in this study. The behavioral data from nine of these subjects were not available due to equipment failure. Of the remaining 39 subjects, their mean age was 43.8 years (± 11.7), and their mean education was 15.4 years (± 2.3), and 15 were women.

### Neuroimaging Acquisition

Neuroimaging data collection began on a 3-Tesla Siemens Allegra scanner (24 subjects) and was completed on a 3-Tesla Siemens Skyra scanner (15 subjects). For this reason, a regressor for scanner was included in all group-level analyses, as has been done in previous research utilizing more than one scanner (Stonnington et al., 2008; Biswal et al., 2010; Wylie et al., 2018). A T2^∗^-weighted echo planar sequence was used to collect functional images during eight blocks (four at each of two difficulty levels), with 140 brain volume acquisitions per block (Allegra: echo time = 30 ms; repetition time = 2,000 ms; field of view = 22 cm; flip angle = 80°; slice thickness = 4 mm, 32 slices, matrix = 64 × 64, in-plane resolution = 3.438 × 3.438 mm^2^; Skyra: echo time = 30 ms; repetition time = 2,000 ms; field of view = 22 cm; flip angle = 90°; slice thickness = 4 mm, 32 slices, matrix = 92 × 92, in-plane resolution = 2.391 × 2.391 mm^2^). A high-resolution magnetization-prepared rapid gradient echo (MPRAGE) image was also acquired (Allegra: TE = 4.38 ms; TR = 2,000 ms, FOV = 220 mm; flip angle = 8°; slice thickness = 1 mm, NEX = 1, matrix = 256 × 256, in-plane resolution = 0.859 × 0.859 mm^2^; Skyra: TE = 3.43 ms; TR = 2,100 ms, FOV = 256 mm; flip angle = 9°; slice thickness = 1 mm, NEX = 1, matrix = 256 × 256, in-plane resolution = 1 × 1 mm^2^) and was used to register the functional data into standard MNI space for group analysis and for the volumetric analyses.

### Behavioral Paradigm and Data

Behavioral data acquisition and stimulus presentation were administered using the E-Prime software (Schneider et al., 2002). During the fMRI scan, participants were presented with the *n*-back working memory task in which task difficulty was varied by presenting the 0-back condition, which places a low load on working memory, and the 2-back condition, which places a higher load on working memory. There were four blocks of each level of the *n*-back task (eight blocks total), with 65 trials per block. The four blocks of each task were always presented together (that is, the two tasks were not interleaved), and the order of presentation (0-back first vs. 2-back first) was counterbalanced across subjects. During the 0-back task (control task), participants were asked to respond each time the target letter “K” was presented on the screen, while during the 2-back task, participants were asked to respond when the target letter corresponded to the letter presented two trials before (e.g., R N Q N…). Letters were presented in white (Arial 72 point font) on a black background. Of the 26 letters in the English alphabet, 10 were excluded to enhance the discriminability of the letters used as stimuli. The following letters were used (with equal frequency): A, B, C, D, F, H, J, K, M, N, P, Q, R, S, T, V, and Z. The letter stimuli remained on the screen for 1.5 s, followed by a 500 ms inter-trial interval (ITI), and the time between successive trials was jittered to allow for the data to be deconvolved as an event-related design. The jittering was optimized using the Optseq2 program^1^. The jittering was achieved by inserting between zero and six null events between successive trials. The duration of each null event was a multiple of the length of the trial (in this case, 2 s), drawn from a distribution following a power function. The majority of inter-trial intervals were 500 ms (zero null events), followed by 2 s (one null event) and so on. The average ITI was 1,587.87 ms (±1,769.7). All subjects practiced both tasks prior to the scanning session.

In order to ensure comparable stimulation across subjects, the stimuli always remained on the screen for 1.5 s (that is, they were not removed when subjects responded), and each run lasted the same amount of time (260 s). The average amount of time between successive blocks was 2 min 04 s (*SD* = 2 min 17 s).

The following behavioral data were analyzed: overall accuracy, which was the number of trials in which the correct response was made divided by the total number of trials, the reaction times (RTs) of the correct trials, and signal detection metrics. Signal detection analysis was used to separate discrimination sensitivity from response bias—factors that can independently affect accuracy (Macmillan and Creelman, 1991; Anderson et al., 2011). The ability to correctly identify target stimuli was measured using the discriminability index (*d*’), calculated as (*z*FA - *z*HR), where *z* is the inverse of the standard normal cumulative distribution, FA is the false-alarm rate (the proportion of responses made to stimuli that were not targets), and HR is the hit rate (the proportion of correct identifications of target stimuli). In the context of this experiment, where all stimuli were readily discernable, *d*’ is best thought of as perceptual certainty rather than as sensitivity to stimulation. Response bias was measured using “criterion” (β), calculated as −1/2(*z*HR + *z*FA) with higher values (fewer false alarms and fewer hits) indicating reduced response bias or more conservative responding. Lower criterion values (more hits and more false alarms) indicated increased response bias and more liberal responding.

### VAS-F

To evaluate the level of on-task or “state” fatigue, participants were presented with a visual analog scale (VAS) before and after each block of the *n*-back task. Participants were asked: “How mentally fatigued are you right now?” and were asked to indicate their level of fatigue on a scale from 0 to 100, with 0 being not fatigued at all and 100 being extremely fatigued. In order to mask the purpose of the study, five additional VASs were administered as well, in randomized order. These assessed happiness, sadness, pain, tension, and anger.

Because VAS-F scores were obtained before and after each run, the amount of fatigue during each block was estimated by using the mean of the scores before and after the relevant block; this value was used in the correlational analyses. Furthermore, because we were specifically interested in cognitive fatigue, we divided the data into blocks on which subjects reported at least some fatigue and blocks on which they reported no fatigue (zero on the VAS-F; see Table 1). This was done because it is reasonable to hypothesize that when at least some fatigue was reported, subjects were engaged in the task and that fatigue-related areas should be active. However, when no fatigue was reported, it is less clear what to hypothesize. This may have represented a failure of introspection, in which case it would be a mistake to attempt to relate the fatigue score to brain activation. Alternatively, it could represent zero fatigue, which might be related to minimal activation (or even deactivation) in fatigue-related areas, or it could represent some combination of these cases. Because of this, we felt it more straightforward to analyze only those data for which we had clear hypotheses. A chi-squared test showed the number of runs with and without fatigue was comparable across the two tasks (χ^2^(1) = 1.40, *p* = 0.24). The blocks on which subjects reported at least some fatigue were used for the main analyses. Finally, because the VAS-F scores were skewed, they were transformed using the Box-Cox method to ensure that assumptions of normality were not violated (Box and Cox, 1964). The Box-Cox method is a power transformation in which a range of power transformations are considered and the one that best transforms the data into a normal distribution is selected.

**TABLE 1:** Number and percentages of runs on which subjects reported no fatigue relative to runs where they reported at least some fatigue, as a function of task (0-back vs. 2-back).

	0-Back	2-Back
Fatigue	115 (76%)	102 (69%)
No fatigue	36 (24%)	45 (31%)

### Analyses

#### RT and Accuracy

Mean RT was calculated using accurate trials. For both the RT and accuracy data, a linear mixed effects [LME; using the R statistical package (version 3.4.3)] was used with the factors of task (0-back vs. 2-back), run (runs 1–4 of each task), and VAS-F (the visual analog scale of fatigue) as a quantitative variable; subject was a random factor.

#### SDT Measures (*d*’ and Bias)

For each of the SDT measures [sensitivity (*d*’) and response bias], an LME was used with the factors of task (0-back vs. 2-back), run (runs 1–4 of each task), and VAS-F (using the same transformed and averaged values as were used for the RT and accuracy analyses), as a quantitative variable and subject was included as a random factor.

#### Neuroimaging

The neuroimaging data was preprocessed using *fMRIPrep* 1.4.1 (Esteban et al., 2019; RRID:SCR_016216), which is based on *Nipype* 1.2.0 (Gorgolewski et al., 2011; RRID:SCR_002502).

#### Anatomical Data Preprocessing

For anatomical preprocessing, the T1-weighted (T1w) image from each subject was corrected for intensity non-uniformity (INU) with N4BiasFieldCorrection (Tustison et al., 2010), distributed with ANTs 2.2.0 (Avants et al., 2008; RRID:SCR_004757), and used as T1w-reference throughout the workflow. The T1w-reference was then skull-stripped with a *Nipype* implementation of the antsBrainExtraction.sh workflow (from ANTs), using OASIS30ANTs as target template. Brain tissue segmentation of cerebrospinal fluid (CSF), white matter (WM), and gray matter (GM) was performed on the brain-extracted T1w using fast (FSL 5.0.9, RRID:SCR_002823, Zhang et al., 2001).

#### Anatomical Normalization

Volume-based spatial normalization to one standard space (MNI152NLin2009cAsym) was performed through non-linear registration with antsRegistration (ANTs 2.2.0), using brain-extracted versions of both T1w reference and the T1w template. The following template was selected for spatial normalization: *ICBM 152 Non-linear Asymmetrical template version 2009c* (Fonov et al., 2009, RRID:SCR_008796; TemplateFlow ID: MNI152NLin2009cAsym).

#### Anatomical Volumetric Calculations

For each subject, the normalized volume of the striate was calculated using the results generated by Freesurfer’s segmentation. Specifically, the volume of the nucleus accumbens, the caudate, and the putamen (bilaterally) were added together and the result was divided by the total intracranial volume. This was used for our volumetric analyses in which we correlated the normalized striatal volume with subjects’ VAS-F, criterion, and *d*’ scores.

#### Functional Data Preprocessing

For functional data preprocessing, the following preprocessing was performed on each of the eight BOLD runs of fMRI data per subject (i.e., four runs of each task). First, a reference volume and its skull-stripped version were generated using a custom methodology of *fMRIPrep*. The BOLD reference was then co-registered to the T1w reference using flirt (FSL 5.0.9, Jenkinson and Smith, 2001) with the boundary-based registration (Greve and Fischl, 2009) cost-function.

#### Co-registration

Co-registration was configured with nine degrees of freedom to account for distortions remaining in the BOLD reference volume. Head-motion parameters with respect to the BOLD reference (transformation matrices and six corresponding rotation and translation parameters) were estimated before any spatiotemporal filtering using mcflirt (FSL 5.0.9, Jenkinson et al., 2002). BOLD runs were slice-time corrected using 3dTshift from AFNI 20160207 (Cox and Hyde, 1997, RRID:SCR_005927).

#### Resampling

The BOLD time-series (including slice-timing correction) were resampled onto their original, native space by applying a single, composite transform to correct for head-motion and susceptibility distortions. These resampled BOLD time-series will be referred to as *preprocessed BOLD in original space*, or just *preprocessed BOLD*. The BOLD time-series were resampled into standard space, generating a *preprocessed BOLD run in MNI space (using the “MNI152NLin2009cAsym” template)*.

#### Confounding Variables

Several confounding time-series were calculated based on the *preprocessed BOLD*: framewise displacement (FD), DVARS, and three region-wise global signals. FD and DVARS are calculated for each functional run, both using their implementations in *Nipype* (following the definitions by Power et al., 2014). The three global signals were extracted within the CSF, the WM, and the whole-brain masks. Additionally, a set of physiological regressors was extracted to allow for component-based noise correction (*CompCor*, Behzadi et al., 2007). Principal components were estimated after high-pass filtering the *preprocessed BOLD* time-series (using a discrete cosine filter with 128 s cut-off) for the two *CompCor* variants: temporal (tCompCor) and anatomical (aCompCor). tCompCor components were then calculated from the top 5% variable voxels within a mask covering the subcortical regions. This subcortical mask was obtained by heavily eroding the brain mask, which ensured that it did not include cortical GM regions. For aCompCor, components were calculated within the intersection of the aforementioned mask and the union of CSF and WM masks calculated in T1w space, after their projection to the native space of each functional run (using the inverse BOLD-to-T1w transformation). Components were also calculated separately within the WM and CSF masks. For each CompCor decomposition, the *k* components with the largest singular values were retained, such that the retained components’ time series were sufficient to explain 50% of variance across the nuisance mask (CSF, WM, combined, or temporal). The remaining components were dropped from consideration. The head-motion estimates calculated in the correction step were also placed within the corresponding confound file. The confound time series derived from head-motion estimates and global signals were expanded with the inclusion of temporal derivatives and quadratic terms for each (Satterthwaite et al., 2013). Frames that exceeded a threshold of 0.5 mm FD or 1.5 standardized DVARS were annotated as motion outliers. The CompCor components, motion parameters, and FD values were included in the deconvolution as regressors of no interest.

#### Interpolation

All resamplings were performed with *a single interpolation step* by composing all the pertinent transformations (i.e., head-motion transform matrices, susceptibility distortion correction when available, and co-registrations to anatomical and output spaces). Gridded (volumetric) resamplings were performed using antsApplyTransforms (ANTs), configured with Lanczos interpolation to minimize the smoothing effects of other kernels (Lanczos, 1964). Non-gridded (surface) resamplings were performed using mri_vol2surf (FreeSurfer).

#### Deconvolution

The resulting data were then deconvolved. In the deconvolution, signal drift was modeled with a set of basis functions; the motion parameters were used as regressors of no interest, and TRs with motion exceeding 1.7 mm (half a voxel, in native space) were excluded from analysis [resulting in the exclusion of an average of 3.8 TRs (2.8%) per subject and an average of 0.5 TRs (0.4%) across the dataset]. The CompCor components and FD values were also included as regressors of no interest. The regressors of interest were the correct trials of each block. Each block was deconvolved separately, and the coefficient of fit of the correct trials was entered into the group-level analysis.

#### Group-Level Analyses

Because correlations were found between *d*’ and VAS-F, criterion and VAS-F, and between *d*’ and criterion (formal analysis described below), three group-level analyses were conducted: one for VAS-F, one for *d*’, and one for criterion. In all cases, an LME was used (3dLME from the AFNI suite of processing tools) with the factors of task (0-back vs. 2-back) and run (runs 1–4 of each task) and with subject included as a random factor. For the analysis of fatigue, the VAS-F scores were included as a quantitative variable. For the analysis of perceptual sensitivity, the *d*’ scores were included as a quantitative variable. For the analysis of bias, the criterion scores (β) were included as a quantitative variable.

The results of these whole-brain analyses were corrected for multiple comparisons by using an individual voxel probability threshold of *p* < 0.001 and a cluster threshold of 13 voxels (voxel dimension = 3 × 3 × 3 mm). Monte Carlo simulations, using 3dClustSim (version AFNI_17.2.16, compile date: Sept 19, 2017), showed this combination to result in a corrected alpha level of *p* < 0.05. Furthermore, because we were specifically interested in the striatum, we also calculated the cluster threshold necessary to correct for multiple comparisons in an area restricted to the nucleus accumbens, the caudate nucleus, and the putamen, based on the anatomical location of these structures. This calculation showed that with an individual voxel probability threshold of *p* < 0.001 and a cluster threshold of three voxels, the corrected alpha level would be *p* < 0.05.

## Results

### RT and Accuracy

For RT, the main effects of task and run were significant with no evidence for an interaction. The main effect of task [*F*(1, 186.5) = 29.10, *p* < 0.0001] was due to subjects responding with longer latencies for the 2-back task (771 ms) than for the 0-back task (615 ms). The main effect of run [*F*(3, 180.8) = 2.97, *p* < 0.05] was due to subjects responding with progressively longer latencies during the first three runs and then faster latencies on the fourth run: 667, 703, 715, and 687 ms for runs 1–4, respectively. Importantly, there was neither an effect of VAS-F nor did VAS-F interact with any of the factors.

For the accuracy data, the main effect of task was significant [*F*(1, 191.6) = 15.64, *p* < 0.0001]. This resulted from greater accuracy on the 0-back task (93.9%) than on the 2-back task (88.8%). No other effects or interactions were significant: as with the analysis of the RT data, there was neither an effect of VAS-F nor did VAS-F interact with any of the factors.

### SDT Measures

#### Preliminary Analysis

We first tested the independence of *d*’ and criterion by analyzing *d*’ as a function of task, criterion, and run using an LME. There was a strong negative relationship between *d*’ and criterion [*F*(1, 131) = 192.39, *p* < 0.0001], showing that *d*’ and criterion were not independent (see Supplementary Figure 4). The coefficient was −1.69, indicating that as subjects’ perceptual certainty (*d*’) increased, they became less conservative in their response bias. Additionally, to ensure that our tasks induced fatigue, we analyzed the VAS-F scores as a function of task and run (also using an LME). The only significant effect in this analysis was that of run [*F*(3, 177.12) = 4.51, *p* < 0.005]. This resulted from subjects reporting increasingly more fatigue across the four runs of the task (runs 1–4: 22.9, 24.0, 27.3, and 27.9, respectively).

#### Analysis of Criterion

**FIGURE 1:**
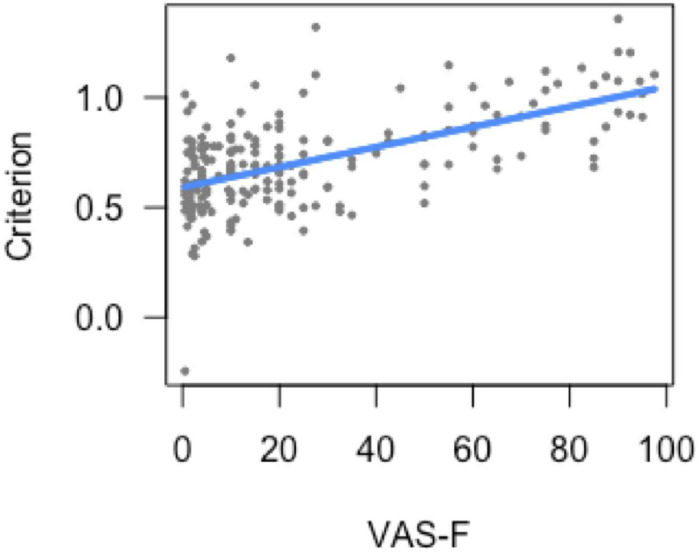
Bias (response criterion) as a function of cognitive fatigue (VAS-F). As cognitive fatigue increased, subjects increased their response criterion. For ease of interpretation, the “raw,” un-transformed VAS-F scores are shown in the plot. VAS-F, visual analog scale of fatigue.

For the analysis of criterion (response bias), there was a main effect of task [*F*(1, 174.5) = 11.56, *p* < 0.001), which was due to a higher criterion (conservative bias) during the 0-back task (0.70) than during the 2-back task (0.60). There was also a significant relationship between criterion and VAS-F [*F*(1, 169.6) = 4.55, *p* < 0.05]. As Figure 1 shows, this was a positive correlation [coefficient (or slope of the linear relationship) = 0.08]: the more fatigue subjects reported, the higher their criterion (i.e., the more conservative their response bias).

#### Analysis of Sensitivity

**FIGURE 2:**
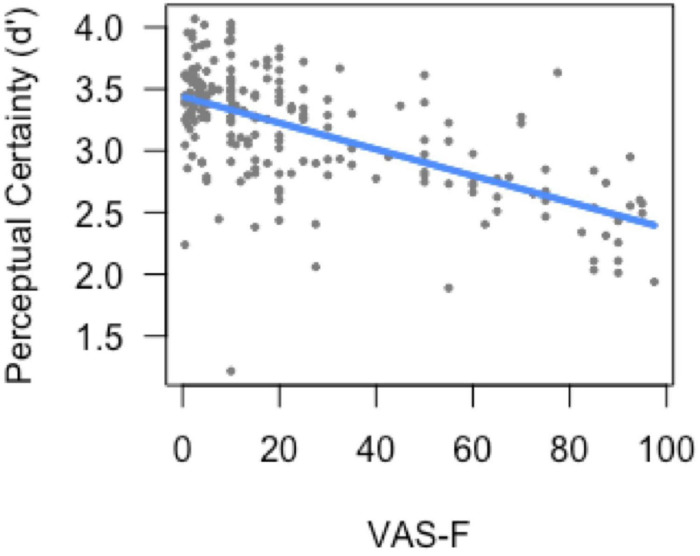
Perceptual certainty (*d*’) as a function of cognitive fatigue (VAS-F). As cognitive fatigue increased, subjects’ perceptual certainty decreased. For ease of interpretation, the “raw,” un-transformed VAS-F scores are shown in the plot. VAS-F, visual analog scale of fatigue.

The analysis of sensitivity (*d*’) showed a main effect of task [*F*(1, 175.3) = 200.97, *p* < 0.001], which was due to higher perceptual certainty (sensitivity) on the 0-back task (3.12) than on the 2-back task (2.22). The main effect of VAS-F was also significant [*F*(1, 147.9) = 3.86, *p* = 0.05]. As Figure 2 shows, this was due to a negative correlation between perceptual certainty and cognitive fatigue scores (coefficient = −0.19): as subjects became more fatigued, their perceptual certainty decreased. No other effects or interactions were significant.

### Structural Neuroimaging Results

We performed three volumetric analyses: we correlated striatal volume with 1) VAS-F, 2) *d*’, and 3) criterion. In all cases, the volumetric data was correlated with the average of the fatigue and SDT measures, which were averaged across task and run (using only those runs where fatigue was reported). To correct for multiple comparisons, we used the Bonferroni approach, in which family-wise errors are corrected by requiring that the *p*-values are less than 0.05/3 (0.017). The correlation between striatal volume and *d*’ was significant (*r* = 0.51, *p* < 0.005), as was the correlation between striatal volume and criterion (*r* = −0.52, *p* < 0.005). However, the correlation between striatal volume and VAS-F was not significant (*r* = −0.27, *p* = 0.13). Because the caudate nucleus has been associated with cognitive fatigue in previous work (Chaudhuri and Behan, 2004; Wylie et al., 2017a), we performed two exploratory analyses in which the volumes of the left and right caudate were correlated with VAS-F. The correlation between VAS-F and the left caudate was not significant (*r* = −0.28, *p* = 0.12), but the correlation between VAS-F and the right caudate did reach conventional levels of significance (*r* = −0.36, *p* < 0.05).

### Functional Neuroimaging Results

In the behavioral analyses above, we found a significant relationship between *d*’ and criterion, as well as a significant relationship between VAS-F and both *d*’ and criterion. Therefore, for the analyses of the neuroimaging data, we performed separate analyses for VAS-F, *d*’, and criterion.

#### Fatigue (VAS-F) Effects

**FIGURE 3:**
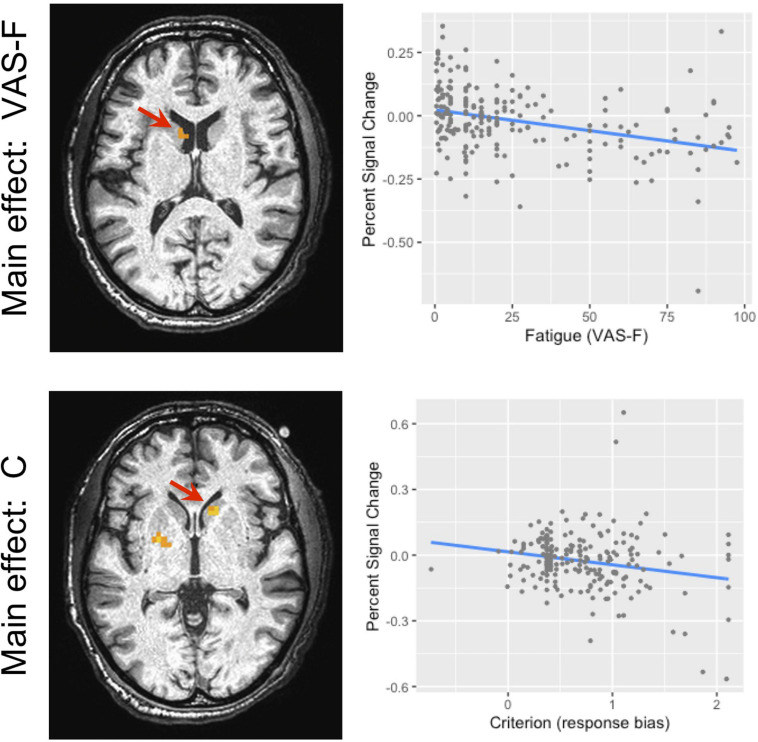
The main effect of fatigue (VAS-F) and criterion in the caudate nucleus of the basal ganglia. Because this was within our region of interest, the cluster level threshold was *k* ≥ 3 voxels. The location of the local maxima of activation is shown in the panels on the left. For the main effect of VAS-F, the location was −7, 9, 14 (*X Y Z*); for the main effect of criterion, the location was 14, 19, −2 (*X Y Z*). The panels on the right show the relationship between brain activation and VAS-F (top) and criterion (bottom). For ease of interpretation, the “raw,” un-transformed VAS-F scores are shown in the plot. VAS-F, visual analog scale of fatigue.

**FIGURE 4:**
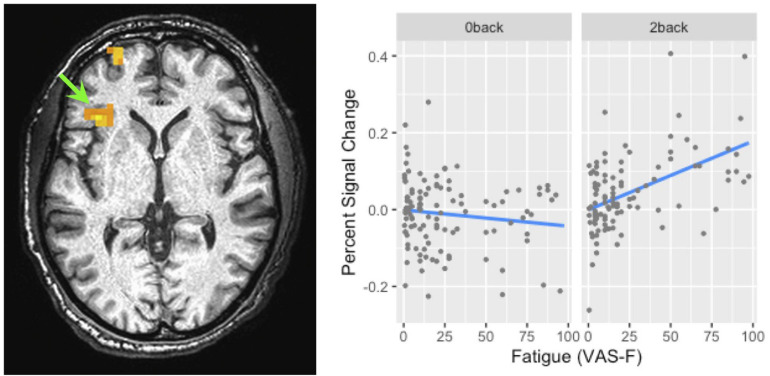
The interaction of task and fatigue (VAS-F) in the insula. The location of this interaction is shown by the green arrow (local maxima *X Y Z*: -34, 26, 6). Because this was not within our region of interest, the cluster level threshold was *k* ≥ 13 voxels. The pattern of the interaction was similar in the superior frontal/orbital gyrus (the location of which can be seen in the left panel). For ease of interpretation, the “raw,” un-transformed VAS-F scores are shown in the plot. VAS-F, visual analog scale of fatigue.

Brain activation correlated with the VAS-F in the caudate of the basal ganglia and the superior frontal gyrus (see Table 2 and Figure 3). Figure 3 shows the negative relationship between the BOLD signal and VAS-F in the caudate (coefficient = −0.047). Furthermore, there was an interaction between task and VAS-F in several frontal areas including the superior frontal gyrus, the insula, and the inferior frontal gyrus (see Table 2). Figure 4 shows the interaction in the insula, which resulted from a negative relationship between the BOLD signal and VAS-F for the 0-back task (coefficient = −0.015) and a positive relationship for the 2-back task (coefficient = 0.050). This pattern was also shown in the superior and inferior frontal gyri.

**TABLE 2:** Fatigue (VAS-F) effects.

Condition/Location	BA	*X*	*Y*	*Z*	Voxels	*F* statistic
**VAS-F**						
Basal ganglia						
Caudate	*–*	−6.6	9.0	14.0	3	11.38
Frontal						
Superior medial gyrus	*10*	−0.1	67.4	10.0	16	17.64

**Task × VAS-F**						
Frontal						
Superior frontal gyrus	*10*	−27.2	64.0	10.0	20	18.94
Insula	*45*	−34.1	26.1	6.0	28	23.13
Inferior frontal gyrus	*11*	41.5	36.5	−10.0	21	17.05

*The brain areas associated with the main effect of VAS-F (top) and with the interaction of task and VAS-F (bottom). BA, Brodmann’s area; X Y Z, the location of the voxel with peak intensity in each cluster; Vox, the number of voxels in the region of overlap.*

#### Criterion Effects

**FIGURE 5:**
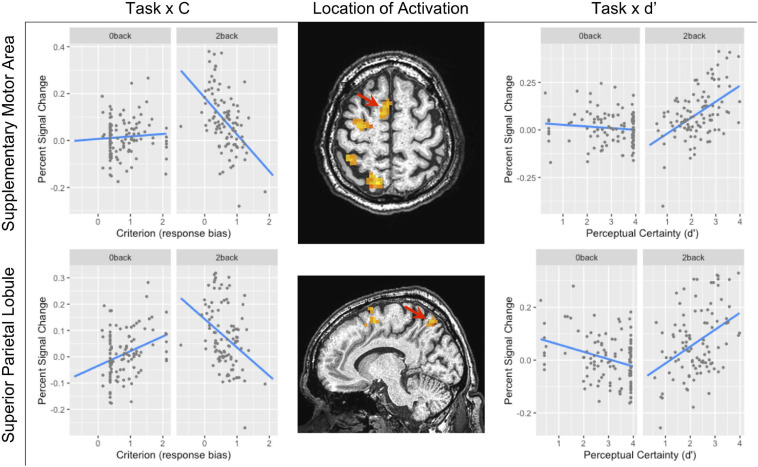
The task × criterion interaction (left column) and task × *d*’ interaction (right column) in the supplementary motor area (SMA) (top row local maxima; *X Y Z*: −7, 2, 62) and in the superior parietal lobule (bottom row local maxima; *X Y Z*: −20, 63, 50). In both rows, the location of the interaction is shown by the red arrow. Because this was not within our region of interest, the cluster level threshold was *k* ≥ 13 voxels.

The BOLD signal correlated with criterion in the caudate and putamen of the basal ganglia (see Table 3 and Figure 3). Figure 3 shows the negative relationship between the BOLD signal and criterion (coefficient = −0.059) in the caudate. There were also interactions between task and criterion in frontal areas [superior orbital and superior frontal gyri, supplementary motor area (SMA), and precentral gyrus], and parietal areas (superior and inferior parietal lobules) (see Table 3). The interaction in the SMA is shown in Figure 5, where the relationship between the BOLD signal and criterion was weakly positive for the 0-back task (coefficient = 0.011), but is strongly negative for the 2-back task (coefficient = −0.157). A similar pattern was shown in the other areas where an interaction was found. For example, Figure 5 shows a similar pattern in the superior parietal lobule: the relationship between the BOLD signal and criterion was positive for the 0-back (coefficient = 0.056) and strongly negative for the 2-back (coefficient = −0.108).

**TABLE 3:** Criterion effects.

Condition/location	BA	*X*	*Y*	*Z*	Voxels	*F* statistic
**Criterion**						
Basal ganglia						
Caudate nucleus	–	14.0	19.3	−2.0	6	14.88
Putamen/thalamus	–	–16.9	–8.2	−6.0	14	14.43

**Task × criterion**						
Frontal						
Superior orbital/frontal gyrus	*10*	24.3	64.0	2.0	16	17.15
Superior frontal gyrus	*6*	–23.8	–1.4	46.0	53	21.09
SMA	*6*	–6.6	2.1	62.0	45	19.90
Precentral gyrus	*6*	–51.3	2.1	50.0	17	18.38
Precentral gyrus	*6*	24.3	–4.8	46.0	14	15.27
Parietal						
Superior parietal lobule	*7*	–20.4	–63.2	50.0	39	19.87
Inferior parietal lobule	*7*	–34.1	–52.9	54.0	37	19.80

*The brain areas associated with the main effect of criterion (top) and with the interaction of task and criterion (bottom). BA, Brodmann’s area; X Y Z, the location of the voxel with peak intensity in each cluster; Vox, the number of voxels in the region of overlap.*

#### *d*’ Effects

There were no areas where there was a main effect of perceptual certainty (*d*’) on the BOLD signal. However, there were interactions between task and perceptual certainty (*d*’) in the putamen of the basal ganglia, frontal areas (SMA and precentral gyrus), and in parietal areas (superior and inferior parietal lobule) (see Table 4). As Figure 5 shows, the relationship between the BOLD signal and *d*’ in the SMA was weakly negative for the 0-back task (coefficient = −0.009), but markedly positive for the 2-back task (coefficient = 0.084). This was also the case in the superior parietal lobule (see Figure 5): the relationship between the BOLD signal and *d*’ was weakly negative for the 0-back task (coefficient = −0.028) and more strongly positive for the 2-back task (coefficient = 0.064).

**TABLE 4:** Perceptual certainty (*d*’) effects.

Condition/Location	BA	*X*	*Y*	*Z*	Voxels	*F* statistic
**Task × *d*’**						
Basal ganglia						
Putamen	*–*	–20.4	–1.4	10.0	6	14.10
Frontal						
SMA	*6*	–6.6	5.5	74.0	37	22.56
Precentral gyrus	*6*	–30.7	–11.7	58.0	30	21.85
Precentral gyrus	*6*	–41.0	–4.8	38.0	13	16.38
Pre/postcentral gyrus	*6*	–58.2	–1.4	22.0	17	21.95
Parietal						
Superior parietal lobule	*7*	–20.4	–63.2	50.0	71	25.29
Inferior parietal lobule	*40*	–44.4	–39.2	46.0	33	20.58

*The brain areas associated with the main effect of d’ (top) and with the interaction of task and d’ (bottom). BA, Brodmann’s area; X Y Z, the location of the voxel with peak intensity in each cluster; Vox, the number of voxels in the region of overlap.*

## Discussion

Previous work has indicated that the two central metrics of SDT—perceptual certainty and criterion—may be related to cognitive fatigue. Perceptual certainty has been shown to decrease after subjects complete a fatiguing task (Matthews and Desmond, 2002), and changes in fatigue have been linked to changes in the effort–reward payoff matrix (Dobryakova et al., 2015; Müller and Apps, 2018). Here, we assessed whether changes in cognitive fatigue correlated with changes in both perceptual certainty and criterion and also how these measures changed as a function of changes in brain activation. Behaviorally, changes in subjects’ VAS-F scores were not correlated with RT or accuracy (see Supplementary Figures 2, 3) but were correlated with both criterion and *d*’, supporting the idea that SDT metrics can be used to better understand subjective cognitive fatigue. The fMRI data also support this idea, inasmuch as activation in the striatum was associated with VAS-F, criterion, and *d*’. Together, these data not only show that these metrics are related but also provide some insight into why they are related.

In the behavioral data, there was a positive relationship between cognitive fatigue and response bias (criterion), such that as subjects reported more fatigue, their response bias became more conservative. When we investigated the areas of the brain that were responsive to cognitive fatigue and to response bias, the striatum was involved in both, though the areas responsive to each did not overlap. Furthermore, the pattern of activation in the striatum associated with cognitive fatigue was comparable to the pattern associated with response bias (see Figure 3). Taken together, these results offer support for the idea that cognitive fatigue is related to response bias.

Additionally, we found that cognitive fatigue was negatively related to perceptual certainty (*d*’). That is, as subjects reported more cognitive fatigue, their perceptual certainty declined. This conforms to everyday experience—when we are fatigued, we feel “less sharp” and less confident in our assessment of our surroundings—and is also consistent with previous findings (Matthews and Desmond, 2002). However, this current result is the first time that changes in cognitive fatigue have been shown to be correlated with changes in *d*’. Furthermore, we also found that the volume of the striatum was related to SDT measures during working memory processing. The relationship was negative for criterion and positive for *d*’, meaning that individuals with greater striatal volume showed a more liberal response criterion and higher perceptual certainty, whereas individuals with a smaller striatum showed a more conservative response criterion and lower perceptual certainty. As in the fMRI results, the directionality of relationship between striatal volume and VAS-F was the same as that between striatal volume and criterion. For the VAS-F, this relationship was not significant when the entire striatal volume was considered. This relationship was significant when only the caudate nucleus was investigated (albeit, only on the right) as motivated by prior research (e.g., Dobryakova et al., 2015, 2018; Wylie et al., 2017a)—further supporting the importance of the caudate nucleus in the experience of cognitive fatigue. Taken together, the volumetric results accord well with the results of the functional neuroimaging data and suggest that cognitive fatigue is related not only to the activation in the caudate of the basal ganglia but also to the volume of the caudate.

More broadly, these findings support our hypothesis that changes in VAS-F would be related to changes in response bias and extend our prediction toward a fuller definition of cognitive fatigue: one of the signatures of cognitive fatigue appears to be a more conservative response bias and lower perceptual certainty. This is seen in the behavioral data and in the relationships between the behavioral data and the BOLD signal. These results also help to explain why simple performance measures such as accuracy often fail to correlate with fatigue: fatigue affects not only the subjects’ ability to distinguish targets from non-targets (*d*’) but also their response bias (criterion). Thus, while subjects’ inability to distinguish targets from non-targets does cause errors, they appear to compensate for this by requiring more evidence before releasing their responses. If one calculates accuracy by averaging across all types of error, this distinction is lost and fatigue-related changes in performance are not evident (a result replicated here in the analysis of the accuracy data, see Supplementary Figure 3). By using SDT on the behavioral data, we are better able to understand the types of performance decrements associated with fatigue; by investigating the associated changes in brain activation, we are able to better understand the mechanisms underlying these changes.

While changes in brain activation in the striatum were associated with both cognitive fatigue and with response bias, the manipulation of task difficulty showed differences in the brain areas associated with cognitive fatigue and SDT measures. For example, in a replication of previous work, we found activation in the insula to be associated with cognitive fatigue (Wylie et al., 2017a; Müller and Apps, 2019). As Figure 4 shows, fatigue-related activation in the insula showed a strong positive relationship to brain activation during the difficult 2-back task and a weaker negative relationship during the easier 0-back task. Finding fatigue-related activation in the insula is consistent with the role of the insula in processing internal states such as fatigue (Müller and Apps, 2019); finding a different relationship between fatigue reported during the two tasks and activation in the insula may suggest that the fatigue experienced during the tasks was qualitatively different. For example, the fatigue experienced during the 0-back task may have been more closely related to boredom (Milyavskaya et al., 2019), whereas the fatigue experience during the 2-back task may have been more closely related to a decrease in the resources necessary to perform the task.

For criterion and *d*’, the manipulation of task difficulty was related to brain areas more closely related to attention and response selection: superior parietal lobule (SPL) and SMA. As Figure 5 shows, both of these areas showed a stronger relationship between SDT metrics and activation during the 2-back than during the 0-back. Furthermore, the relationship between brain activation and criterion and *d*’ were reciprocal. That is, as activation in the SPL and SMA increased during the 2-back, subjects showed increased perceptual certainty and adopted a more liberal response bias. This was not the case during the 0-back task, which is likely due to the fact that the 0-back task is sufficiently easy that relatively small changes in brain activation had little effect on perceptual sensitivity and response bias.

### Limitations and Future Directions

While we did support our hypotheses, our results are nevertheless currently limited by having been demonstrated using only the *n*-back task. It will be important to show that comparable results are found using different tasks. Furthermore, these results should be replicated in a larger sample. While our sample is relatively large, it is still difficult to generalize to the entire population based on approximately 40 healthy individuals. Moreover, having a larger sample would potentially allow us to tease apart the separate effects of VAS-F, criterion, and *d*’ (which were correlated with one another in this sample) through stratifying the sample or performing mediation analyses. Going forward, it will be valuable to determine if these new metrics of cognitive fatigue are sufficiently sensitive to distinguish cognitive fatigue in neurotypical individuals from clinical populations that are particularly affected by fatigue (e.g., individuals with MS or TBI). Additionally, while we favor an interpretation of these data in terms of effort and reward, it is important to point out that reward was not explicitly manipulated in this experiment. The tasks likely differed in their reward value (e.g., the 2-back task was far more difficult than the 0-back task, and good performance on the 2-back was therefore likely to have been more implicitly rewarding than good performance on the 0-back task), but future work should manipulate reward explicitly to test this interpretation more directly.

## Conclusion

The results presented here show that cognitive fatigue is related to changes in subjects’ response bias (payoff matrix) and perceptual certainty. Not only are self-report metrics (VAS-F) related to these SDT metrics but also the striatum is sensitive to all three. These results may suggest that as cognitive fatigue increases, subjects make more errors because their perceptual sensitivity declines and they compensate for this by adopting a more conservative response bias. The mechanisms underlying these changes include brain areas associated with effort and reward (the striatum), attentional processes (fronto-parietal areas), and areas related to response conflict (SMA).

## Data Availability Statement

The data analyzed in this study is subject to the following licenses/restrictions: Kessler Foundation reserves the rights to this dataset. Requests to access these datasets should be directed to GW, gwylie@kesslerfoundation.org.

## Ethics Statement

The studies involving human participants were reviewed and approved by the Kessler Foundation Institutional Review Board. The patients/participants provided their written informed consent to participate in this study.

## Author Contributions

GW and JD conceived of the study concept. GW oversaw data collection, performed the analyses, and drafted the manuscript. BY, JS, and JD provided critical revisions. All authors approved the final version of the manuscript for submission.

## Conflict of Interest

The authors declare that the research was conducted in the absence of any commercial or financial relationships that could be construed as a potential conflict of interest.

## Footnotes

1 https://surfer.nmr.mgh.harvard.edu/optseq/
