# Supplementary material for: Using Signal Detection Theory to Better Understand Cognitive Fatigue
Source: Front Psychol. 2021 Jan 15;11:579188. doi: 10.3389/fpsyg.2020.579188 (PMC7844088; doi:10.3389/fpsyg.2020.579188)
Supplement: Supplementary file 1 [file Data_Sheet_1.PDF]

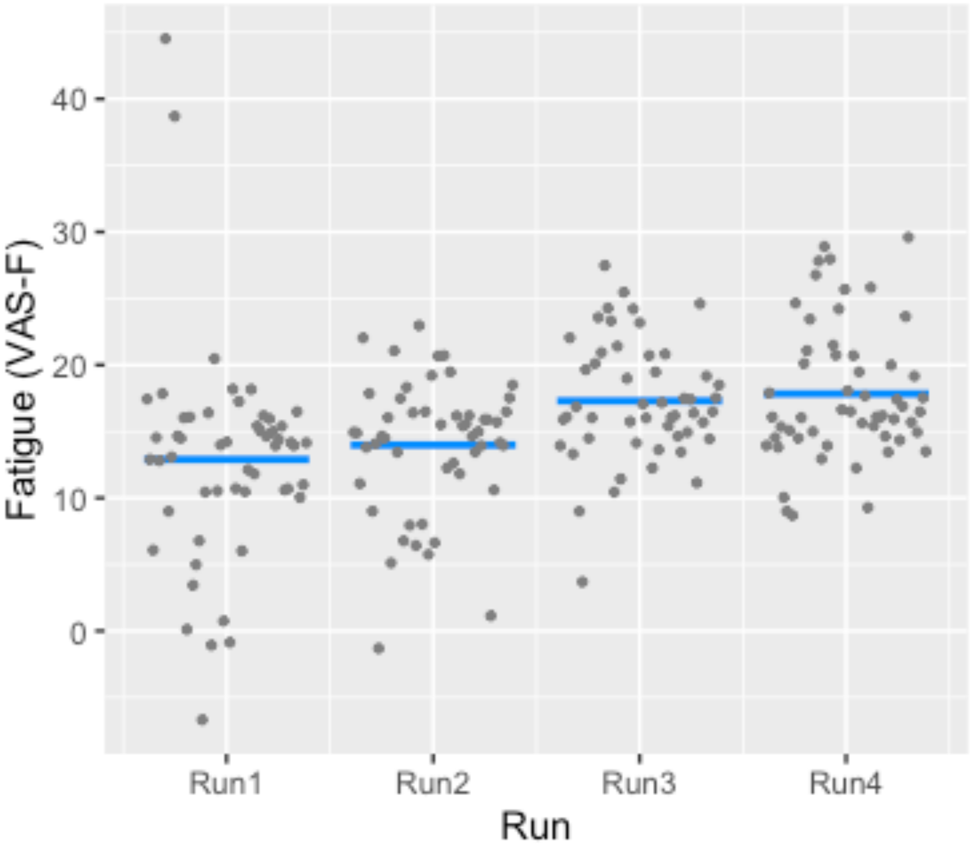

Figure S1. Cognitive fatigue (VAS-F) as a function of Run. Cognitive fatigue increased across the four runs of the two tasks. Note: for ease of interpretation, the 'raw', un-transformed VAS-F scores are shown in the plot.

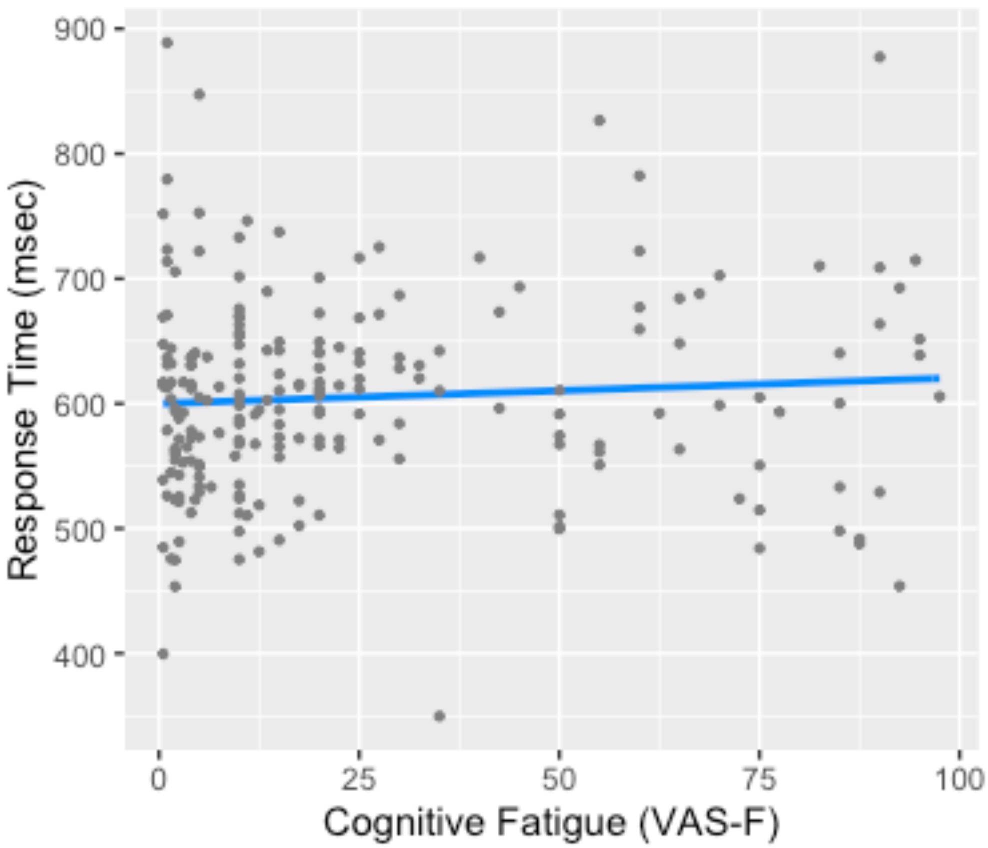

Figure S2. Response time (RT) as a function of Cognitive fatigue (VAS-F). There was not a significant relationship between RT and VAS-F in the data presented here, as shown by the nearly horizontal trend line (blue). Note: for ease of interpretation, the 'raw', un-transformed VAS-F scores are shown in the plot.

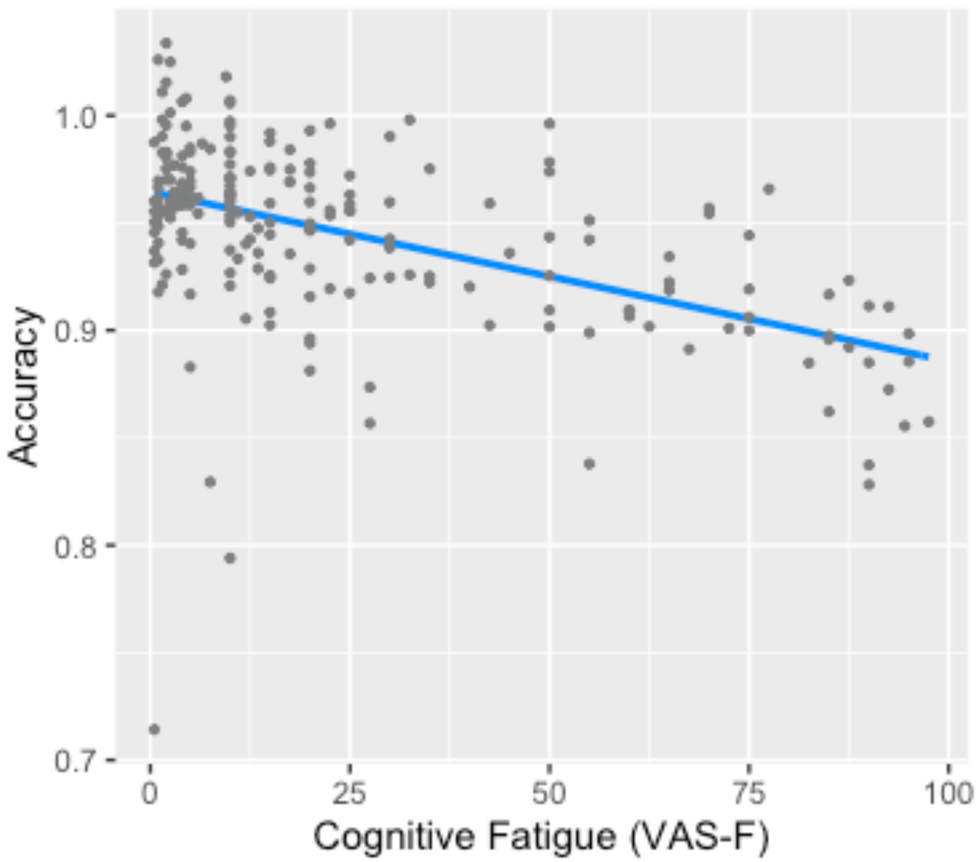

Figure S3. Accuracy as a function of Cognitive fatigue (VAS-F). There was not a significant relationship between accuracy and VAS-F in the data presented here, as shown by the nearly horizontal trend line (blue). Note: for ease of interpretation, the ‘raw’, un-transformed VAS-F scores are shown in the plot.

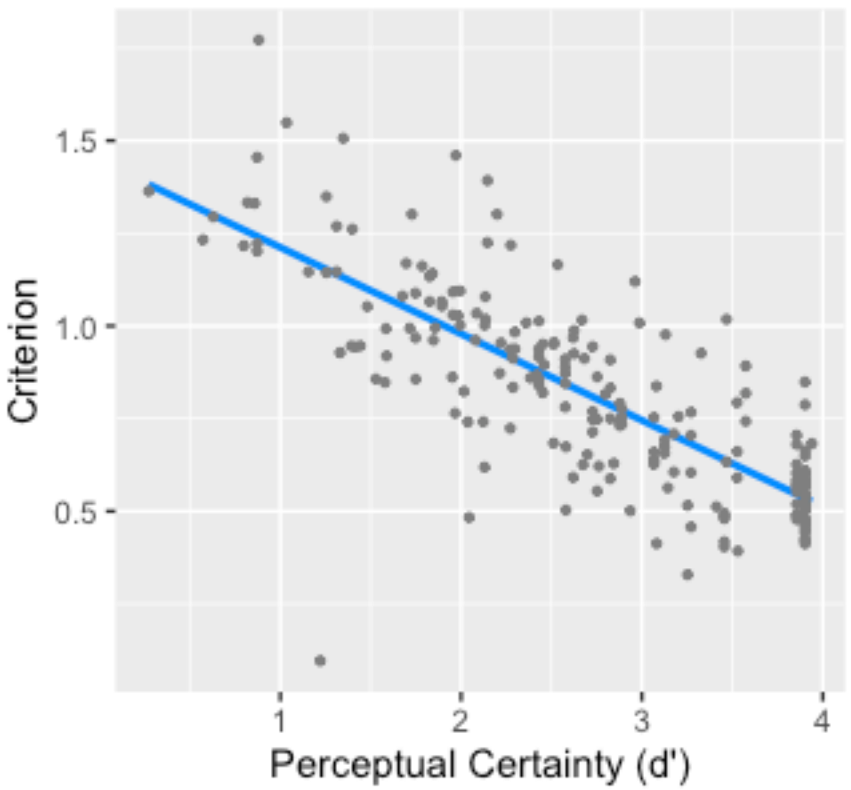

Figure S4. Criterion as a function of perceptual certainty (d’). There was a significant relationship between Criterion and d’ in the data presented here, as shown by the trend line (blue).
